# Factors Affecting Recurrence in 165 Patients with Serous Borderline Ovarian Tumours: The Pattern of Micro-Invasion Is Main Prognostic Factor

**DOI:** 10.3390/jcm14062050

**Published:** 2025-03-18

**Authors:** Zehra Ozturk Basarır, Tufan Arslanca, Yesim Ozkaya Ucar, Sevgi Ayhan, Bülent Ozdal

**Affiliations:** Department of Gynecologic Oncology, Ankara City Hospital, Ankara 06800, Turkey; drtufanarslanca@hotmail.com (T.A.); yesimozkaya@gmail.com (Y.O.U.); sevgiserdar@hotmail.com (S.A.); bulentozdal@yahoo.com (B.O.)

**Keywords:** micro-invasion, recurrence, serous borderline ovarian tumour

## Abstract

**Background**: The aim of this study was to evaluate the serous borderline ovarian tumours (BOTs), the recurrence rates, and the factors affecting recurrence. **Methods**: A total of 165 patients diagnosed with serous BOT between 2004 and 2019 were included. The patients were evaluated in respect of age, preoperative CA125 levels, FIGO stage, tumour size, stromal micro-invasion, the presence of non-invasive implants, surgical procedures, and lymphadenectomy performed, or all that affects disease-free survival. **Results**: Early-stage BOT (stage I–II) was determined in 149 (90.3%) patients. Conservative surgery was performed in 57 (34.5%) patients. The non-invasive implantation was detected in 19 (11.5%) patients, and micro-invasion was determined in 31 (18.8%) patients. The median follow-up was 120 months, and recurrence was observed in 8 (4.8%) patients. The 5-year disease-free survival rate was 95.2%, and the 10-year disease-free survival rate was also 95.2%. Univariate analysis showed that elevated preoperative CA125 levels and the presence of micro-invasion were associated with poor disease-free survival outcomes. In the multivariate analysis, the presence of micro-invasion was the only independent poor prognostic factor (HR: 8.944, 95%CI: 2.060–38.833; p:0.003). **Conclusions**: The micro-invasion was the main factor for recurrence in patients with serous BOT.

## 1. Introduction

Borderline ovarian tumours (BOTs) constitute approximately 10–20% of epithelial ovarian tumours [1]. These tumours, which have a clinical course between benign and malignant, were previously defined by the International Federation of Gynaecology and Obstetrics (FIGO) as tumours with low malignant potential and most recently have been classified as atypical proliferative tumours in the World Health Organisation (WHO) 2020 classification [2,3].

Although BOTs share some histological and clinical characteristics, such as extrapelvic spread, nuclear atypia, and mitotic activity similar to epithelial ovarian cancers, they can be differentiated, as there is no clear destructive stromal invasion [4]. However, they do show the capability for micro-invasion (largest linear diameter <5 mm) into the stroma in one or more foci. The spread pattern has been seen to be extensive, and BOTs also show the characteristic of the ability for lymph node involvement and non-invasive implantation on peritoneal surfaces [5,6]. Serous BOTs are the most prevalent type, comprising approximately 50% of all BOTs. The majority of serous BOTs are diagnosed at a young age and at an early stage and thus have a good prognosis, unlike ovarian cancers [7]. The recurrence of serous BOTs has been observed in 5–8% of cases, manifesting as serous BOT or low-grade serous carcinoma, which has a negative impact on patient prognosis. The following sites of recurrence have been reported for BOTs: the peritoneum, pelvic lymph nodes, and the lung/pericardium, especially the remaining ovarian tissue. International guidelines recommend unilateral salpingo-oophorectomy and multiple peritoneal biopsies for patients with childbearing potential who wish to preserve their fertility. Minimally invasive or ultra-minimally invasive approaches can be applied to this patient subgroup. While the literature indicates that reproductive outcomes following conservative treatment are favourable, a higher rate of recurrence has been documented in patients undergoing fertility-sparing surgery in contrast to radical treatments. Furthermore, a higher rate of progression to cancer has been observed in BOTs following conservative surgery [8]. The recurrence rate is influenced by several factors, including advanced stage, bilateral tumours, micro-invasion, non-invasive implant, and the surgical treatment type. However, recurrence can also be seen in patients with no known risk factors, and it has been reported in multicentre studies of large series wherein differences in the surgical techniques applied can affect the outcomes. Although fertility-sparing surgery is accepted in the treatment of borderline ovarian tumours, the National Comprehensive Cancer Network (NCCN) guidelines delineate comprehensive surgery as encompassing hysterectomy, bilateral salpingo-oophorectomy, omentectomy, peritoneal biopsies, and peritoneal washings. In accordance with these guidelines, hysterectomy is regarded as a component of the standard primary surgical management of BOTs [9].

In this relatively young group of patients, it is important to identify the high-risk group with a high likelihood of recurrence. It is also important to know these patients in order to decide how to follow them, how long and how often to contact them, and to offer them additional treatment options, taking into account their desire for fertility [10,11].

The aim of this single-centre study was to describe the recurrence rates in patients with serous BOTs in relation to clinical, pathological, and surgical characteristics and the prognostic risk factors affecting recurrence. Furthermore, this study sought to identify the predictive value of factors associated with the potential reappearance of serious BOTs in patients of childbearing potential who were treated conservatively.

## 2. Materials and Methods

The single-centre study included 165 patients who had been diagnosed with serous BOT pathologically in the Gynaecological Oncology Department of Zekai Tahir Burak Training and Research Hospital in Ankara, Turkey between 2004 and 2019. In the present institution, which functions as a tertiary hospital specialising in women’s health and oncology surgery, a total of 451 cases of BOT patients who underwent surgical intervention were recorded during the specified period. However, subsequent exclusions were necessary, as 286 patients were deemed ineligible for various reasons. These exclusions included cases where patients were concurrently diagnosed with a secondary malignancy, where data was incomplete, where the diagnosis was non-serous BOT, or where patients did not adhere to regular follow-up appointments. Following the application of exclusion criteria, the study’s final sample comprised 165 patients diagnosed with serous BOTs. Approval for the study was granted by the Institutional Ethics Committee (decision no: E2-23-4163, dated: 26 May 2023). Written informed consent for the use of their clinical data was provided by all the patients.

The following data were collected from the patient’s files and medical records: age, parity, preoperative CA125 IU/mL levels, FIGO stage, tumour bilaterality, tumour size (cm), the presence of stromal micro-invasion, the presence of non-invasive implant, the type of surgery, whether lymphadenectomy was performed, follow-up, and recurrence time.

The serous BOT stage, as documented in the patients’ pathology reports, was revised according to the 2021 FIGO staging system [12]. In our gynaecological oncology unit, a frozen section procedure is routinely performed. All patients are asked about their fertility wishes during the preoperative counselling process. All patients diagnosed with BOTs underwent a conservative or a definitive surgery, depending on their fertility wishes. Conservative surgery was defined as a surgery which left the uterus and a part of at least one of the ovaries intact, and the removal of both ovaries and the uterus was defined as a definitive surgery.

Patients diagnosed with serous BOTs in the gynaecology department and referred to the gynaecological oncology clinic were evaluated by the multidisciplinary gynaecological oncology council, and the decision for re-operation and surgical staging was performed according to the age and desire for fertility of the patient and the previously applied surgery. As part of the surgical-staging procedure, peritoneal washing, multiple peritoneal biopsies, omentectomy, and/or lymph node dissection (sampling/systematic) were performed. Omental or peritoneal implants that were not found to be invasive were classified as non-invasive implants. Invasive implants, which were accepted as low-grade serous carcinoma according to the 2020 WHO classification [3], were excluded from the study.

The surgical specimens were subsequently subjected to evaluation by a team of pathologists specialising in the field of gynaecological oncology. The patients were invited to attend regular polyclinic follow-up examinations at six-month intervals for the first five years and then on an annual basis. The follow-up programme encompassed pelvic examinations, hemogram, blood biochemistry, and transabdominal and transvaginal/pelvic ultrasonography. Patients with suspected recurrence were also assessed using advanced imaging techniques such as thoracoabdominal tomography. Disease-free survival (DFS) was defined as the time from surgery to the first detection of a recurrence or last contact.

### Statistical Analysis

The data obtained in the study were analysed statistically using SPSS v. 24 software (Statistical Package for the Social Sciences). Descriptive statistical methods were employed to evaluate the data, and the results were expressed as mean ± standard deviation (SD), median, minimum, and maximum values, or as numbers (n) and percentages (%). The Shapiro–Wilk test and graphical examinations were employed to assess the conformity of quantitative data to a normal distribution. In instances where two groups of quantitative data exhibited a normal distribution, the Student’s *t*-test was employed. Conversely, if the distribution was deemed to be non-normal, the Mann–Whitney U test was implemented. The Pearson Chi-square test, Fisher’s exact test, and the Fisher–Freeman–Halton test were used in the comparisons of qualitative data. The relationships between quantitative variables were evaluated using Spearman’s correlation analysis. Univariate and multivariate Cox proportional hazards regression analyses were used to determine factors with an effect on recurrence. Survival analysis was performed using the Kaplan–Meier method. A value of *p* < 0.05 was accepted as the level of statistical significance.

## 3. Results

### 3.1. Clinicopathological Characteristics

The study evaluated a total of 165 patients, with a median age of 40 years (range, 17–78 years). The clinicopathological characteristics and laboratory results of the patients are shown in Table 1. Early-stage BOT (stage I–II) was determined in 149 patients (90.3%), while advanced stage BOT (stage III-IV) was determined in 16 patients (9.7%). The median tumour size was 10.8 cm (range, 3–22.5 cm). Furthermore, the presence of bilateral BOTs was observed in 27 (16%) patients. The median preoperative CA125 level was 24 IU/mL (range, 5–2544 IU/mL). Conservative surgery was performed in 57 (34.5%) patients. Lymphadenectomy was performed in 159 (96.4%) patients, resulting in a median total number of 54 lymph nodes removed (range, 19–108). Fifteen (9%) patients underwent a re-staging procedure following the initial operation. Non-invasive implantation was observed in 19 (11.5%) patients, and micro-invasion was identified in 31 (18.8%) patients (see Table 1 for details).

### 3.2. Micro-Invasion

A comparison of the patient demographic data, including age, parity, and tumour size, revealed no statistically significant disparities when stratified by tumour invasion status. The statistical analysis revealed a significant correlation between the CA125 values and the micro-invasion cases, with higher values indicating a greater propensity for invasion (*p* = 0.001; *p* < 0.01). Furthermore, a statistical significance was observed in the association between invasion and implantation status, with cases demonstrating implantation exhibiting a higher rate of invasion compared to those without implantation (*p* = 0.001). Concurrently, the rate of invasion in patients at an advanced stage was found to be statistically significantly higher than in patients at an early stage (*p* = 0.001) (Table 2).

Following the evaluation of the effects of the variables with univariate effects on invasion with the logistic regression model, it was determined that the model was significant (F = 59.97; *p* < 0.01) and that its explanatory power was 90.3%. Within the model, the effects of CA125 and advanced stage were found to be statistically significant (*p* < 0.05). The risk of invasion was found to be 5.322 (95% CI: 1.763–16.067) times and 8.300 (95% CI: 2.279–30.235) times higher, respectively, if CA125 was 34 IU/mL and above. The findings indicate that CA125 and advanced stage are independent risk factors affecting the risk of invasion.

The ROC curve analysis determined a cut-off point of 34 for CA125, at which the sensitivity, specificity, positive predictive value, and negative predictive value were 70.97%, 79.10%, 44%, and 92.20%, respectively. The ROC curve obtained revealed an underlying area of 83.4% and a standard error of 4.1%. A statistically significant correlation was found between invasion and the cut-off value of CA125 (*p* = 0.001; *p* < 0.01). This finding suggests that patients with elevated CA125 levels, specifically those above 34 IU/mL, have an approximately 11-fold increased probability of demonstrating invasion. The odds ratio for CA125 was determined to be 11.392 (95% CI: 4.482–28.952). The results of the diagnostic screening tests and the ROC curve for CA125 for invasion are shown in Figure S1.

### 3.3. Recurrence

The median follow-up period was 120 months and the 5-year DFS was determined to be 95.2%, with the 10-year DFS also recorded as 95.2%. During the follow-up period, recurrence was observed in 8 (4.8%) patients. The time to recurrence ranged from 13 to 60 months. All recurrences were classified as BOTs on pathology, and no patients were lost to the disease (Table 3).

The age range of patients experiencing recurrence was from 18 to 55 years, and 62.5% of them had a CA125 level above 35 IU/mL. Furthermore, 50% of patients underwent definitive surgery, while the remaining 50% underwent conservative surgery. All patients had undergone lymphadenectomy, and 75% had early-stage disease. Moreover, 62.5% of patients exhibited micro-invasion, while only one patient with a recurrence had a non-invasive implant. No statistically significant difference was determined between the surgical groups in respect of age, implant presence, tumour stage, and recurrence rates. Additionally, the analysis revealed no statistically significant difference in recurrence rates between patients who underwent lymphadenectomy and those who did not (see Table 3 for details).

The univariate analysis revealed no statistically significant associations between tumour size and recurrence status (*p* > 0.05) or between age distribution and number of parities. Furthermore, advanced stage disease, non-invasive implants, the presence or absence of lymphadenectomy, and the type of operation were found not to be associated with recurrence. The preoperative median CA125 level was determined to be 62.5 IU/mL (range, 24–230 IU/mL) in patients with recurrence and 22 (range, 5–2544 IU/mL) in patients without recurrence (*p* = 0.006). The micro-invasion group also had a statistically higher recurrence rate (16.1% vs. 2.2%; *p* = 0.006) (Table 4). The multivariate analysis was performed with CA125 and micro-invasion (Table 5). Micro-invasion was determined to be the only independent poor prognostic factor for recurrence. In patients with micro-invasion, hazard ratio (HR) for the recurrence rate was 8.944 (95% CI:2.060–38.833, *p* = 0.003). The DFS graph according to the presence of invasion is given in Figure S2.

## 4. Discussion

Serous BOTs, which are an intermediate form of ovarian tumours, are less common than invasive cancers and have a good prognosis. The aim of this study was to evaluate the prognostic risk factors affecting recurrence. The study results showed that recurrence was determined in 4.8% of patients, that the 5-year DFS was 95.2%, and that the 10-year DFS was 95.2%. The presence of stromal micro-invasion was identified as the only significant independent poor prognostic factor for recurrence.

The recurrence rates of serous BOTs have been documented to range from 5 to 18% according to the tumour characteristics [13,14]. Age, advanced stage, type of surgery, the presence of non-invasive implants, micro-invasion, time to recurrence, and residual disease have been reported as risk factors for recurrence. Although BOTs are known to be tumours with a low malignant potential, their recurrence has a detrimental effect on both the patient’s prognosis and their quality of life [15]. Given that surgery is the primary treatment modality, the type of surgery performed is a crucial factor in determining disease recurrence. In addition, as diagnosis is made before the age of 40 years in approximately 1 in 3 patients, a fertility-preserving surgery is preferred for patients who wish to remain fertile and do not want to experience the complaints of early menopause symptoms [10].

The oncological outcomes and recurrence rates with respect to fertility-preserving surgery and definitive surgery are not clear in previous studies. In a study of 212 patients by Gouy et al., DFS was reported to be statistically significantly reduced in those who underwent a conservative surgery (*p* < 0.001). However, given that the study group exclusively comprised cases involving cystectomy and invasive implants (accepted as LGSOC in the revised terminology), the outcomes may be attributed to the heterogeneity of the study group [16]. A meta-analysis of 260 studies by Vasconcelos et al. reported no statistically significant difference in recurrence rates between groups treated with a conservative surgery of unilateral salpingo-oopherectomy (USO) and those who underwent a definitive surgery. However, the risk of recurrence was found to be statistically significantly higher in those who underwent cystectomy compared with USO (*p* < 0.001) [17]. The results of the current study showed that there was no difference between a conservative surgery and a definitive surgery with respect to recurrence (p:0.449). The rationale underlying this phenomenon can be attributed to the fact that all patients in the conservative surgery group underwent USO while those with invasive implants were excluded from the study. The selection of the patient group according to the new classification and the uniformity of the group are significant strengths of the study.

A subject of debate in the literature is whether or not lymph node sampling should be performed in surgical treatment to reduce recurrence. In a meta-analysis by Seidman et al., the 6-year survival rate was found to be 98% in a group with lymph node involvement in a study that included >4000 patients, and lymph node sampling was reported to have a low prognostic value [18]. Qian et al. also determined that lymph node involvement did not increase the risk of recurrence. Moreover, the 5-year DFS rates were not determined to be statistically different between cases with lymph node positivity and negativity (p:0.078) or between cases with more or less than 10 lymph nodes removed (p:0.549) [11]. In contrast, Üreyen et al. reported a relationship between lymph node involvement in patients with serous BOTs and significantly decreased DFS results [19]. The results of the current study showed no statistically significant difference in the recurrence rates between the patients with and without lymph node removal and the number of lymph nodes.

There is considerable variability in the reported incidence of stromal micro-invasion. An incidence of around 10–50% of serous BOTs has been reported [20]. In the present study, an incidence of micro-invasion of 18% was found. Another subject of debate is the impact of micro-invasion in serous BOTs on oncological outcomes, and the extent of this remains unclear. Some studies have reported that there is no adverse effect on the prognosis [19]. However, Longacre et al. determined micro-invasion in 28 (10.1%) of 276 patients with serous BOTs and defined stromal micro-invasion as an independent poor prognostic factor for overall survival [20]. Similarly, in a study presented by Buttin et al., micro-invasion in serous BOTs was found to be a poor prognostic factor for recurrence and survival [21]. The results of our study showed that micro-invasion was a single, independent risk factor for recurrence, supporting the findings of the above-mentioned studies. The presence of micro-invasion was found to be associated with an 8944-fold increase in the probability of recurrence (95% CI: 2.060–38.833, *p* = 0.003).

In the 2014 and 2020 WHO classification, non-invasive implants are classified as serous BOTs. In the new updated terminology, invasive implants are defined as low-grade ovarian carcinoma [2,3]. Therefore, studies conducted after 2014 are more relevant [22]. In the current study, cases with pathology results of an invasive implant were evaluated as low-grade serous carcinoma and were excluded from the study. In a study by Hannibal et al., implants were classified as non-invasive and invasive in a study, and it was reported that the risk of recurrence and serous carcinoma development was only statistically significant in the non-invasive implant group [23]. A similar finding was reported in a study by Vang et al. who stated that the presence of a non-invasive implant was a poor prognostic factor for recurrence in a group of 867 atypical proliferative borderline tumours [24]. Conversely, in the study by McKenney et al., no significant correlation was found between non-invasive implants and survival or recurrence [25]. The results of the current study showed that non-invasive implants, as defined, did not increase the risk of recurrence. However, non-invasive implants were found to be statistically significantly higher in patients with microinvasion (*p* = 0.001).

The utilisation of CA125 in the diagnosis of BOTs is a contentious issue. A high CA125 level is of low diagnostic value for the histology and in the differential diagnosis with invasive malignancies. However, it has been observed to contribute to the diagnosis of serous BOTs, in comparison to other borderline types, and it is recommended that if the preoperative level is high, it should be used in the follow-up of postoperative recurrence [26,27]. The results of the current study showed that the risk of recurrence was statistically significantly higher in the group with a high preoperative CA125 level.

The retrospective design and limited number of patients are considered to be the limitations of the study. Despite these limitations, the strengths of the study include the inclusion of patients treated by gynaecological oncologists in a national reference centre hospital with a standardised treatment protocol and regular long-term follow-up. The patient cohort in this study exhibited homogeneity in accordance with the novel WHO classification.

In conclusion, it is imperative to identify a high-risk group of patients in order to prevent recurrence in BOTs. In this study, we identified that in patients with serous BOTs who are determined to have micro-invasion, there is a high risk of recurrence. The presence of micro-invasion was found to be the only poor prognostic factor for recurrence, having an almost 9-fold increased risk. Additionally, this study revealed that elevated CA125 levels increased the risk of micro-invasion. Consequently, it is recommended to refer patients to a specialist surgical oncology centre in order to optimise and harmonise the overall management of these patients. However, further prospective studies are still needed to definitively demonstrate the importance of micro-invasion.

## Figures and Tables

**Table 1:** Distribution of the descriptive characteristics of the patients.

Characteristics		
Age	Mean ± SD	41.72 ± 14.21
	Median (Min–Max)	40 (17–78)
Tumour Size	Mean ± SD	11.06 ± 3.96
	Median (Min–Max)	10.8 (3–22.5)
CA125	Mean ± SD	78.85 ± 266.63
	Median (Min–Max)	24 (5–2544)
		**n (%)**
Micro-invasion	Absent	134 (81.2%)
	Present	31 (18.8%)
Operation Type	Definitive ^1^	108 (65.5%)
	Conservative ^2^	57 (34.5%)
Lymphadenectomy	Not performed	6 (3.6%)
	Performed	159 (96.4%)
Non-invasive Implant ^3^	Absent	146 (88.5%)
	Present	19 (11.5%)
Re-staging	Not performed	150 (91%)
	Performed	15 (9%)
Stage	Early stage (I–II)	149 (90.3%)
	Advanced stage (III–IV)	16 (9.7%)

1: Definitive surgery: removal of both ovaries and the uterus. 2: Conservative surgery: defined as the preservation of the uterus and at least one or part of the ovaries. 3: Peritoneal and/or omental. SD: Standard Deviation.

**Table 2:** The presence of invasion according to descriptive characteristics.

Characteristics		Invasion		*p*
		Negative	Positive	
Age	Mean ± SD	41.52 ± 13.86	42.58 ± 15.83	^c^ 0.710
	Median (Min–Max)	40 (18–78)	45 (17–77)	
Tumour size	Mean ± SD	11.13 ± 4.05	10.76 ± 3.61	^a^ 0.632
	Median (Min–Max)	10.5 (3–22.5)	11 (5.9–20))	
CA125	Mean ± SD	50.79 ± 223.21	200.13 ± 386.05	^a^ 0.001 **
	Median (Min–Max)	22 (5–2544)	65 (8–1810)	
Paritiy	Absent	21 (67.7%)	10 (32.3%)	^d^ 0.259
	Present	113 (84.3%)	21 (15.6%)	
Operation	Definitive ^1^	86 (79.6)	22 (20.4)	^d^ 0.474
	Conservative ^2^	48 (84.2)	9 (15.8)	
Non-invasive Implant ^3^	Absent	127 (87.0)	19 (13.0)	^b^ 0.001 **
	Present	7 (36.8)	12 (63.2)	
Stage	Early stage (I-II)	131 (87.9)	18 (12.1)	^b^ 0.001 **
	Advanced stage (III-IV)	3 (18.8)	13 (81.3)	

a: Mann–Whitney U test. b: Fisher’s exact test. c: Student’s *t*-test. d: Pearson Chi-Square test ** *p* < 0.01. 1: Definitive surgery: removal of both ovaries and the uterus. 2: Conservative surgery: defined as the preservation of the uterus and at least one or part of the ovaries. 3: Peritoneal and/or omental, SD: Standard Deviation.

**Table 3:** Analysis of 8 patients with recurrence.

Patient No	Age	Tumour Size (cm)	CA125 (IU/mL)	Operation Type ^1^	Lymphadenectomy	Non-Invasive Implant ^2^	Stage	Micro-Invasion	Time to Recurrence (Month)
1	32	9	41	Definitive	Performed	Present	IIb	Absent	48
2	55	20	210	Definitive	Performed	Absent	IIIb	Present	13
3	22	7	230	Conservative	Performed	Absent	Ic	Present	60
4	55	12	84	Definitive	Performed	Absent	Ic	Present	37
5	37	7	166	Definitive	Performed	Absent	IIIb	Present	48
6	18	8	24	Conservative	Performed	Absent	Ic	Present	24
7	18	18	27	Conservative	Performed	Absent	Ic	Absent	22
8	29	15	33	Conservative	Performed	Absent	Ia	Absent	19

1: Operation Type: definitive surgery: removal of both ovaries and the uterus. Conservative surgery: defined as the preservation of the uterus and at least one or part of the ovaries. 2: Peritoneal and/or omental.

**Table 4:** The development of recurrence according to the descriptive characteristics.

Characteristics		Recurrence		*p*
		Negative	Positive	
Age	Mean ± SD	42.15 ± 14.08	33.25 ± 14.98	^a^ 0.081
	Median (Min–Max)	42 (17–78)	30.5 (18–55)	
Tumour size	Mean ± SD	11.00 ± 3.91	12.15 ± 5.00	^a^ 0.632
	Median (Min–Max)	10.8 (3–22.5)	10.5 (7–20)	
CA125	Mean ± SD	77.68 ± 272.71	101.88 ± 86.73	^a^ 0.006 **
	Median (Min-Max)	22 (5–2544)	62.5 (24–230)	
Micro-invasion	Absent	131 (97.8%)	3 (2.2%)	^b^ 0.006 **
	Present	26 (83.9%)	5 (16.1%)	
Operation	Definitive ^1^	104 (96.3%)	4 (3.7%)	^b^ 0.449
	Conservative ^2^	53 (93.0%)	4 (7.0%)	
Lymphadenectomy	Not performed	6 (100.0%)	0 (0.0%)	^b^ 1.000
	Performed	151 (95.0%)	8 (5.0%)	
Non-invasive Implant ^3^	Absent	139 (95.2%)	7 (4.8%)	^b^ 1.000
	Present	1 8 (94.7%)	1 (5.3%)	
Stage	Early stage (I-II)	143 (96.0%)	6 (4.0%)	^b^ 0.175
	Advanced stage (III-IV)	14 (87.5%)	2 (12.5%)	

a: Mann–Whitney U test. b: Fisher’s exact test ** *p* < 0.01. 1: Definitive surgery: removal of both ovaries and the uterus. 2: Conservative surgery: defined as the preservation of the uterus and at least one or part of the ovaries. 3: Peritoneal and/or omental. SD: Standard Deviation.

**Table 5:** Multivariate analysis of the risk factors for recurrence.

Factors	Recurrence		Multivariate	
	Absent	Present	HR (95% CI)	*p*
**CA125**				
Mean ± SD	77.68 ± 272.71	101.88 ± 86.73	1.000 (0.997–1.002)	0.697
Median (Min–Max)	23 (5–2544)	62.5 (24–230)		
**Micro-invasion**				
Absent	131 (97.8)	3 (2.2)	Reference	0.003 **
Present	26 (83.9)	5 (16.1)	8.944 (2.060–38.833)	

** *p* < 0.01.

## Data Availability

The original contributions presented in this study are included in the article/Supplementary Materials. Further inquiries can be directed to the corresponding author(s).

## Supplementary Materials

The following supporting information can be downloaded at: https://www.mdpi.com/article/10.3390/jcm14062050/s1, Figure S1: ROC curve Analysis for Ca125 in Relation to Micro-invasion; Figure S2: Disease Free Survival Presence of Micro-invasion.
