# Supplementary material for: Factors Affecting Recurrence in 165 Patients with Serous Borderline Ovarian Tumours: The Pattern of Micro-Invasion Is Main Prognostic Factor
Source: J Clin Med. 2025 Mar 18;14(6):2050. doi: 10.3390/jcm14062050 (PMC11942785; doi:10.3390/jcm14062050)
Supplement: Supplementary file 1 [file jcm-14-02050-s001.zip › jcm-3461252-supplementary.pdf]

		Diagnostic Scan				ROC Curve		
	Cut off	Sensitivite	Spesifisite	Positive Predictive Value	Negative Predictive Value	Area	95% Confidence Interval	*p*
**Ca125**	*≥34*	70.97	79.10	44.00	92.20	**0.834**	0.754-0.915	***0.001***

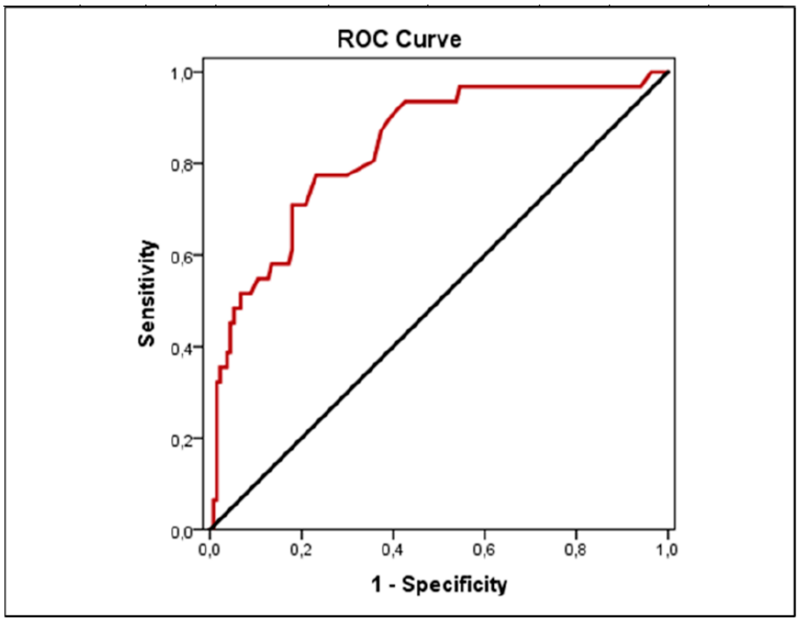

Figure S1. ROC curve Analysis for Ca125 in Relation to Micro-invasion.

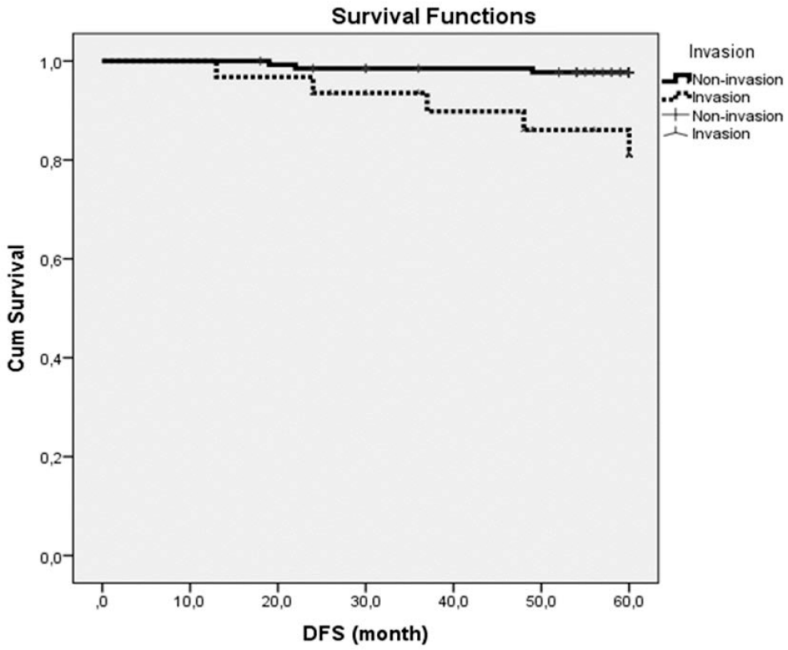

Figure S2. Disease Free Survival Presence of Micro-invasion.
